# Investigating Constraints Along the Plant Secretory Pathway to Improve Production of a SARS-CoV-2 Spike Vaccine Candidate

**DOI:** 10.3389/fpls.2021.798822

**Published:** 2022-01-04

**Authors:** Emmanuel Margolin, Matthew Verbeek, Warren de Moor, Ros Chapman, Ann Meyers, Georgia Schäfer, Anna-Lise Williamson, Edward Rybicki

**Affiliations:** ^1^Division of Medical Virology, Department of Pathology, Faculty of Health Sciences, University of Cape Town, Cape Town, South Africa; ^2^Wellcome Trust Centre for Infectious Disease Research in Africa, University of Cape Town, Cape Town, South Africa; ^3^Faculty of Health Sciences, Institute of Infectious Disease and Molecular Medicine, University of Cape Town, Cape Town, South Africa; ^4^Biopharming Research Unit, Department of Molecular and Cell Biology, Faculty of Health Sciences, University of Cape Town, Cape Town, South Africa; ^5^International Centre for Genetic Engineering and Biotechnology, Observatory, Cape Town, South Africa

**Keywords:** glycoprotein, vaccine, chaperone, processing, protease, degradation, molecular farming

## Abstract

Given the complex maturation requirements of viral glycoproteins and the challenge they often pose for expression in plants, the identification of host constraints precluding their efficient production is a priority for the molecular farming of vaccines. Building on previous work to improve viral glycoprotein production in plants, we investigated the production of a soluble SARS-CoV-2 spike comprising the ectopic portion of the glycoprotein. This was successfully transiently expressed in *N. benthamiana* by co-expressing the human lectin-binding chaperone calreticulin, which substantially increased the accumulation of the glycoprotein. The spike was mostly unprocessed unless the protease furin was co-expressed which resulted in highly efficient processing of the glycoprotein. Co-expression of several broad-spectrum protease inhibitors did not improve accumulation of the protein any further. The protein was successfully purified by affinity chromatography and gel filtration, although the purified product was heterogenous and the yields were low. Immunogenicity of the antigen was tested in BALB/c mice, and cellular and antibody responses were elicited after low dose inoculation with the adjuvanted protein. This work constitutes an important proof-of-concept for host plant engineering in the context of rapid vaccine development for SARS-CoV-2 and other emerging viruses.

## Introduction

The absence of suitable infrastructure to produce vaccines for emerging viruses leaves most developing countries vulnerable and unable to respond appropriately to emerging disease threats. This was first highlighted during the 2009 H1N1 pandemic and remains a major challenge to this day, as evidenced by the ongoing SARS-CoV-2 pandemic (Mortimer et al., 2012). Although several highly effective vaccines have already been approved for use, the infrastructure to facilitate local production is almost entirely absent in Africa (Margolin et al., 2020a). Unsurprisingly, this has culminated in an unacceptable reliance on high income countries and charitable initiatives, such as COVAX, to support the roll out of vaccines to prevent COVID-19 (Adepoju, 2021). Furthermore, implementation of these vaccines has been hindered by high purchasing costs, limitations in global production capacity (Wouters et al., 2021), as well as stringent storage requirements for certain vaccines which are impractical in under-resourced areas (Margolin et al., 2020a).

In the absence of widespread immunity to SARS-CoV-2, uncontrolled viral circulation has given rise to novel variants which rapidly predominate in vulnerable populations (Tegally et al., 2021a,b). In the extreme, these variants may partly undermine natural immunity from previous infections or even vaccine-elicited immunity (Cele et al., 2021; Madhi et al., 2021), although pre-existing immunity in either scenario still confers considerable clinical benefit. Nonetheless, the emergence of novel viral variants adds to the burden of morbidity and mortality in resource-limited areas where vaccine coverage is limited and could also potentially jeopardize global efforts to contain the pandemic if vaccine-elicited immunity does not confer adequate cross-protection (Fontanet et al., 2021).

These scenarios have culminated in renewed interest in establishing end-to-end vaccine manufacturing in Africa where most countries are poorly equipped to contend with the ongoing pandemic (Margolin et al., 2020a). While this is not expected to impact the outcome of COVID-19 in the short term, establishing independent vaccine production capabilities will be critical to responding to future outbreaks or for provision of seasonal vaccines. This will require considerable capital investment and will need to focus on technology platforms that are suited to the African context (Margolin et al., 2020a). Ideally, such a manufacturing platform would need to be cost-effective given the economic constraints on the continent, rapidly scalable to enable timeous response to a pandemic outbreak, and amenable to large scale manufacturing to support widespread implementation on the continent. Accordingly, we are pursuing the development of a plant-based expression platform for SARS-CoV-2 and other vaccines which offer the potential for lower cost manufacturing, rapid expansion of biomass for manufacturing, and lower capital outlay to establish a good manufacturing practice (GMP)-compliant facility compared to conventional mammalian cell production platforms (Murad et al., 2020).

GMP-compliant plant-based biologics manufacturing facilities have already been established in high income countries (Holtz et al., 2015), and several promising vaccines are under clinical development (Ward et al., 2020; Kurokawa et al., 2021), including a virus-like particle (VLP) vaccine for SARS-CoV-2 based on the spike glycoprotein, or S, which is currently in the final stages of efficacy assessment (NCT05040789 and NCT04636697; Ward et al., 2021). This vaccine, developed by Medicago Inc., comprises of the extracellular region of the spike glycoprotein fused to the transmembrane and cytoplasmic regions of influenza hemagglutinin (Ward et al., 2021). A tobacco mosaic virus-like particle presenting a spike-derived antigen from Kentucky Bioprocessing is also undergoing early stage clinical testing (NCT04473690) and a plant-derived receptor binding domain from BAIYA Phytopharm has recently entered into Phase 1 testing (NCT04953078). The establishment of a comparable facility in Africa would be an important step toward lessening the reliance of the continent on high income countries for essential pharmaceuticals and would also enable the development of appropriate capacity for pandemic preparedness. Accordingly, we are developing a suite of expression technologies to support the production of complex viral glycoprotein vaccines which are often inefficiently produced in plants (Margolin et al., 2020c, 2021).

The basis for our expression platform is to engineer the plant biofactory to support the maturation of viral glycoproteins along the secretory pathway (Margolin et al., 2020d). This enables host constraints to be addressed by the transient co-expression of elements of the mammalian cellular machinery (Margolin et al., 2020c). Using these approaches, we aim to develop a modular expression platform that enables the production of well glycosylated and appropriately folded viral glycoprotein-based vaccines that will enable timeous vaccine production in response to a pandemic outbreak. Accordingly, we have shown that the co-expression of human chaperones (Protein origami^™^) enables the production of heavily glycosylated viral glycoproteins in plants which would otherwise only accumulate at very low levels: this approach was validated by the successful production of soluble HIV-1 gp140 trimers at far higher levels than was previously possible and this has shown similar promise for other prototype viral glycoproteins (Margolin et al., 2020b). This has also been combined with the co-expression of human furin to support proteolytic processing *in planta*, where this would not otherwise occur (Margolin et al., 2020c). The impetus behind these approaches is to produce recombinant viral glycoproteins in as close to their native conformation as possible and while many promising plant-derived SARS-CoV-2 vaccine candidates have been reported these have mostly focused on the receptor binding domain (Rattanapisit et al., 2020; Maharjan et al., 2021; Mamedov et al., 2021; Shin et al., 2021) or chimaeric spike-derived antigens (Ward et al., 2021) rather than a native spike that recapitulates the virion-associated glycoprotein structure.

Additional constraints in the host glycosylation machinery are also being investigated in order to determine how they impact glycoprotein production and their relevance to vaccine development (Margolin et al., 2021). Lastly, the impact of the plant protease repertoire on viral glycoprotein production in plants is also unknown but given the abundance of proteases along the secretory pathway, there are concerns that this could negatively impact glycoprotein accumulation (Jutras et al., 2020; Margolin et al., 2020d). The emergence of SARS-CoV-2 and the ensuing global pandemic provided a real world case study of an emerging virus where these host engineering approaches could be implemented for the development of a candidate vaccine to test the utility of our host engineering platform. Accordingly, we systematically investigated constraints along the plant secretory pathway to improve the production of a candidate SARS-CoV-2 vaccine, which we then tested for immunogenicity in mice.

## Materials and Methods

### Design of a Soluble Spike Mimetic for Expression in Plants

A soluble derivative of the SARS-CoV-2 spike was designed based on the first publicly available genetic sequence (GenBank accession: MN908947.3). The coding region was truncated to remove the transmembrane and cytoplasmic domains of the glycoprotein. The native leader sequence was replaced with the tissue plasminogen activator (TPA) signal peptide and the putative furin recognition sequence (RRAR) was replaced with a hexa-arginine (RRRRRR) motif to promote proteolytic processing. A GCN4 fibritin trimerization foldon domain was incorporated at the end of the gene sequence, preceded by a flexible linker peptide (GSGSGS). A polyhistidine (HHHHHH) affinity tag was added to the C-terminus of the antigen after a second linker (GSGGSGGSG). The antigen coding sequence was synthesized by GenScript to reflect the preferred human codon usage and synthetic restriction sites were added to the 5′ and 3′ termini of the DNA. The gene sequence was cloned into pEAQ-*HT*, using AgeI and XhoI, for expression in plants (Sainsbury et al., 2009).

### Generation of Recombinant *A. tumefaciens* Strains

Recombinant pEAQ-*HT* expressing SARS-CoV-2 S∆TM was transformed into *A. tumefaciens* AGL1 as previously described (Margolin et al., 2020c). *A. tumefaciens* AGL1 strains encoding human chaperones and furin were reported in previous studies (Margolin et al., 2020c). Expression plasmids for NbPot1, NbPR4, and HsTIMP were developed in a previous study (Grosse-Holz et al., 2018) and were transformed into *A. tumefaciens* GV3101.

### Small-Scale Expression Screens

Protein expression in plants was conducted as previously described using three plants per group for each experimental repeat (Margolin et al., 2020c). In the case where multiple proteins were co-expressed, equal amounts of the relevant bacterial cultures were mixed resulting in a final OD_600_ of 0.5 each. Crude plant homogenate was harvested 5 days post-infiltration, using Tris-buffered saline (pH 7.5) for extraction, as published previously (Margolin et al., 2019). Crude lysate was quantified using the *DC* protein assay and adjusted to equal amounts of total soluble protein using phosphate-buffered saline (pH 7.4) as a diluent. All screening experiments were performed at least twice and promising approaches were further validated.

### Detection of Recombinant Spike Protein Expression

Samples containing the antigen of interest were resolved on SDS-PAGE gels and then immunoblotted using established procedures (Margolin et al., 2019, 2020c). The recombinant spike was detected with mouse monoclonal anti-histidine antibody (Serotech, MCA1396), diluted 1:2000, which in turn was detected using 1:5000 diluted goat anti-mouse IgG alkaline phosphatase conjugate (Sigma, A3562).

### Large Scale Protein Production in Plants

Batches of up to 100 plants were co-infiltrated with *A. tumefaciens* strains encoding SARS-CoV-2 S∆TM and CRT, each diluted to a final OD_600_ of 0.5. Agroinfiltrated leaves were harvested 5 days post-agroinfiltration and either processed immediately or stored at −80°C. Biomass was homogenized in 2 buffer volumes of Tris-buffered saline (pH 7.5), supplemented with Depol^™^ 40 l (Biocatalysts) and cOmplete^™^, EDTA-free Protease Inhibitor Cocktail (Merck). The homogenate was incubated for 1 h at 4°C, with shaking, to promote maceration of the cell wall and recovery of the protein of interest. Insoluble debris was removed by filtering the sample through Miracloth (Merck) and the resulting sample was clarified by centrifugation at 17000 g. The homogenate was then filtered through a 0.45 μM Stericup^®^ filter unit (Merck) and the recombinant protein was purified using *Galanthus nivalis* lectin affinity chromatography and gel filtration in accordance with previous reports (Margolin et al., 2020c, 2021). Pooled fractions from size-exclusion chromatography were further concentrated using a Vivaspin^®^ centrifugal concentrator with a 30 kDa molecular weight cut-off. The purified protein was resolved by BN-PAGE and quantified as described in published methods (Margolin et al., 2019).

### Mouse Immunizations

Mouse immunizations were conducted at the University of Cape Town’s Animal Research Facility in the Faculty of Health Sciences. All procedures were carried out in accordance with established protocols that were ratified by the UCT animal ethics committee (AEC 020_024). Female BALB/c mice (>8 weeks old) were communally housed in TYPE 2 long cages and acclimatized to their environment for 10 days prior to any experimental procedures. Groups of five mice were randomly distributed into experimental and placebo groups. Animals were immunized with 3 μg of purified protein, formulated 1:1 in Alhydrogel^®^, by intramuscular injection into the tibialis muscle on day 0, 21, and 42. Blood was drawn from the tail artery on days 0, 14, 35, and 56. Mice were sacrificed on day 56 by exsanguination.

### Serum Antibody Binding Elisa

Antibody binding ELISAs were conducted using pooled serum samples for each group. The experiment was conducted as previously described, unless indicated otherwise below (Margolin et al., 2019). ELISA plates were coated with recombinant *E. coli*-produced SARS-CoV-2 spike protein (Invitrogen, RP-87668) to eliminate any potential bias. Experimental time points in the control group which failed to yield a quantifiable endpoint titer were attributed an arbitrary value of 10 to enable the data to be plotted. Polyclonal goat anti-mouse IgG HRP (Abcam, 97,023) was used to detect bound murine IgG which recognized the coating antigen. Data were analyzed and presented as described previously (Margolin et al., 2019).

### IFN-γ ELISpot

The frequency of antigen-specific T cells was determined at the experimental endpoint by IFN-γ ELISpot. Briefly, 96-well plates were coated with 100 μl per well of 5 μg/ml anti-mouse IFN-γ antibody (BD Pharmingen) and incubated overnight at 4°C. The plates were then blocked for 2 h in R10 media [RPMI 1640, 1% Pen/Strep, 2 mM L-Glutamine, 100 μl 50 mM 2-ME, and 10% CTL-Test^™^ medium (CTL Immunogen)] at room temperature. Freshly harvested splenocytes were isolated from each mouse by mashing the spleen through a metal sieve strainer, with a 5 ml syringe plunger, into RPMI media. The splenocytes were washed three times with RPMI media and then lysed for 1 min by the addition of 1 ml Red Blood Cell (RBC) lysis buffer (Sigma R7757). Splenocytes were counted and 5 × 10^5^ cells were added to each well of the 96-well plate. This was followed by the addition of either 100 μl of concanavalin A (1 μg/ml), spike peptide pools (2 μg/ml; GenScript: RP30020), irrelevant peptide (2 μg/ml), or RPMI media (blank) to the plate in triplicate. The plate was incubated overnight at 37°C and then sequentially washed three times each with 100 μl of H_2_O and PBST. The biotinylated detection antibody (BD Pharmingen) was added at 2 μg/ml and incubated for 2 hours in the dark. The plate was then washed three times with PBST and 100 μl of Avidin-horseradish peroxidase solution (BD Pharmingen) was added to the plate for an hour. Finally, the plate was washed 3× each with 100 μl PBST and then PBS before detecting with Nova Red substrate solution. The reaction was terminated by rinsing with tap water. The plate was dried overnight and the number of Spot Forming Units (SFU) was determined using a CTL Immunospot ELISpot reader.

### Pseudovirus Neutralization Assay

Murine plasma from the terminal bleed was evaluated for SARS-CoV-2 neutralizing activity against the matched virus using a pseudovirus neutralization assay. Single-cycle SARS-CoV-2 pseudovirions, based on the HIV backbone, were generated by co-transfection of plasmids pNL4-3.Luc.R-.E- (aidsreagent #3418) and pcDNA3.3-SARS-CoV-2-spike Δ18 into HEK-293TT cells (Rogers et al., 2020). Cell culture supernatants containing the virions were harvested 3 days post-transfection and incubated with heat-inactivated murine plasma at 5-fold serial dilutions for 60 min at 37°C. Plasma/pseudovirus mixtures were then used for transfection of HEK-293 T cells stably expressing the ACE2 receptor (Mou et al., 2020). Cells were lysed 3 days post-infection using the Cell Culture Lysis Reagent (Promega) and assessed for luciferase activity using a GloMax^®^ Explorer Multimode Microplate Reader (Promega) together with the Luciferase assay system (Promega). The half maximal inhibitory dilution (ID50) of each tested plasma sample was calculated in GraphPad Prism using a Non-linear regression (Ferrara and Temperton, 2018).

## Results

### Design of a Soluble SARS-CoV-2 Spike Antigen

**Figure 1:**
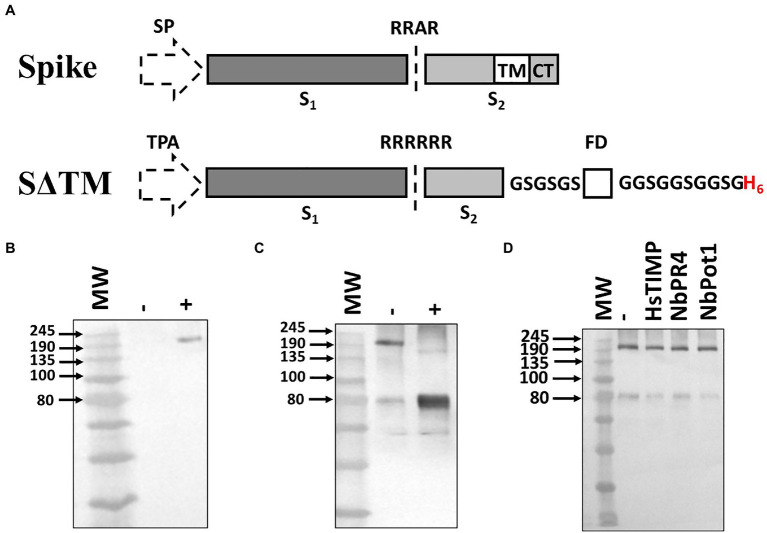
Design and expression optimization of SARS-CoV-2 S∆TM in *N. benthamiana*. **(A)** Schematic of the synthetic gene sequence encoding the S∆TM antigen and the parental (wildtype) sequence from which it was derived (Spike). The native signal peptide (SP) was replaced with a heterologous leader sequence from tissue plasminogen activator (TPA). The furin cleavage sequence (RRAR) was replaced with a hexa-arginine motif (RRRRRR) to enhance processing. A GCN4 fibritin trimerization foldon domain (FD) and a His tag (H_6_) were included at the C-terminus following GSGSGS and GGSGGSGGSG linkers, respectively. The locations of transmembrane region (TM), cytoplasmic tail (CT), and S_1_ and S_2_ domains of the spike are indicated. **(B)** Western blot showing expression of S∆TM alone (−) and with human calreticulin, CRT (+). **(C)** Western blot demonstrating co-expression of S∆TM with CRT and human furin to demonstrate integration of expression approaches. A control where the glycoprotein was co-expressed with CRT but not furin (−) was included alongside the experimental sample (+). **(D)** Western blot demonstrating co-expression of S∆TM with broad-spectrum protease inhibitors HsTIMP, NbPR4, and Nbpot1. In each case, the antigen was co-expressed with CRT and the protease inhibitor of interest. A negative control was included where the glycoprotein was co-expressed with CRT only (−). MW, molecular weight marker.

A soluble spike antigen (S∆TM), consisting of both the S1 and S2 subunits but lacking the transmembrane and cytoplasmic domains, was designed for optimal expression and processing in plants (Figure 1A). The native viral leader sequence was replaced with the tissue plasminogen activator leader sequence (TPA) to promote translocation of the antigen into the secretory pathway. The modified spike included an optimized cleavage sequence (RRRRRR) to promote processing by furin, as previously reported for the HIV envelope glycoprotein when produced in both plants and mammalian cells (Binley et al., 2002; Margolin et al., 2020c). A GCN4 fibritin trimerization domain and a His tag were added to the C-terminus of the antigen, following a flexible glycine-rich linker sequence. No further stabilizing mutations were included in the synthetic antigen as these were only reported subsequent to the initiation of the study (Wrapp et al., 2020).

### Host Engineering to Support SARS-CoV-2 Spike Production

Building on previous work, we implemented a series of different expression strategies to address potential host constraints for SARS-CoV-2 spike production. Firstly, we investigated the co-expression of human calreticulin which has been reported to improve the accumulation of similarly complex glycoproteins in plants (Margolin et al., 2020c). The co-expression of the chaperone substantially improved the accumulation of the antigen, as the glycoprotein was undetectable under the conditions tested when the chaperone was not present (Figure 1B). This is consistent with our previous work suggesting that the plant chaperone machinery may not support efficient folding of complex viral glycoproteins (Margolin et al., 2020c). Based on this data, all subsequent expression screens included the co-expressed chaperone.

Despite the inclusion of an enhanced furin cleavage site, the antigen was not efficiently processed in plants unless the human protease was co-expressed (Figure 1C). Following furin co-expression, the predominant product detected was the ~80 kDa cleavage fragment generated from processing at the interface of the S_1_ and S_2_ subunits (Walls et al., 2020). This suggests that the combination of the co-expressed protease and the enhanced cleavage site supports highly efficient processing in plants, providing a useful approach to produce processed cleaved glycoproteins in plants where this may be required for protein folding. We also tested the combination of co-expressing calreticulin with several prototype broad-spectrum protease inhibitors to determine if they had an additive effect. Considerable evidence has shown that endogenous plant proteases can impair the accumulation of recombinant antibodies in plants reducing yields and leading to unwanted cleavage (Hehle et al., 2015; Puchol Tarazona et al., 2021). It is plausible that a similar effect could be exerted on viral glycoproteins trafficking through the secretory pathway, although this has not yet been reported. Recently, three novel protease inhibitors (NbPR4 and NbPot1 from *N. benthamiana* and human HsTIMP) were reported to enhance the accumulation of several model glycoproteins (Grosse-Holz et al., 2018). We therefore explored the impact of co-expressing these previously validated expression constructs with CRT and the S∆TM antigen (Figure 1D). However, no obvious improvement was observed following the co-expression of any of these constructs and they were not pursued further.

### Purification of SARS-CoV-2 S∆TM

**Figure 2:**
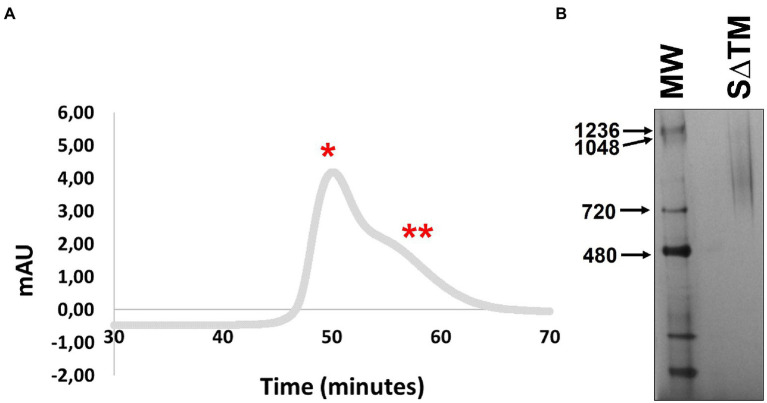
Purification of SARS-CoV-2 S∆TM from plants. **(A)** Gel filtration of affinity-captured antigen using a Superdex 200 HiLoad 16/600 column. The two most prominent peaks are indicated by ^*^ and ^**^, respectively. **(B)** Coomassie-stained BN-PAGE gel containing purified S∆TM.

Following the successful small-scale expression of the SARS-CoV-2 S∆TM antigen, protein production was scaled up. During scale-up furin was not co-expressed as work published subsequent to commencing the study suggested that proteolytic maturation of the spike was not necessary for inducing protective immunity against SARS-CoV-2 (Corbett et al., 2020). Furthermore, the co-expression of the protease could potentially complicate recovery of the glycoprotein if the association between the subunits was labile. Initial attempts to capture the antigen using HisPur^™^ Cobalt resin were unsuccessful. Instead, the glycoprotein was purified by *Galanthus nivalis* lectin affinity chromatography followed by gel filtration, as has been described for other comparable vaccine antigens (Margolin et al., 2019, 2021). The gel filtration profile comprised two broad overlapping peaks which were not well resolved (Figure 2A). Based on previous work producing HIV-1 Env and Marburg virus-derived glycoproteins, aggregated protein would be expected to elute in the first peak, whereas the second peak would contain trimeric protein if present (Margolin et al., 2019, 2021; van Diepen et al., 2019). The fractions comprising the second peak were pooled and concentrated, and then resolved by BN-PAGE. Coomassie staining of the resolved protein yielded a similarly diffuse signal for material derived from peak 2 and no convincing evidence of trimers was observed which would be expected to present as a band ~720 kDa (Figure 2B). This has been observed previously for other plant-derived viral glycoproteins where aberrant glycosylation was associated with agreggation and poor resolution of the purified material by gel filtration or BN-PAGE (Margolin et al., 2021). The recovery of the purified antigen was 65.9 μg/kg (standard deviation = 19.5, *n* = 4).

### Immunogenicity of SARS-CoV-2 S∆TM in Mice

**Figure 3:**
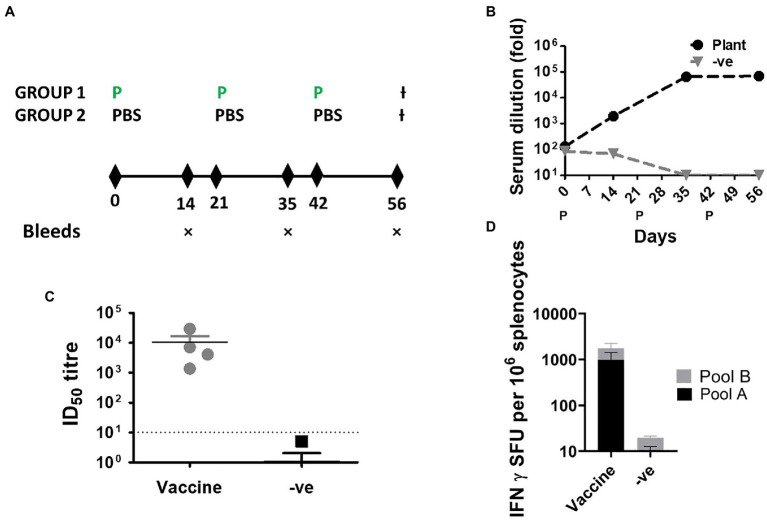
Immunogenicity of SARS-CoV-2 S∆TM in BALB/c mice. **(A)** Schematic of the timing of immunizations and blood draws. Animals were inoculated with the purified antigen on days 0, 21, and 42. Blood was drawn on days 14, 35, and 56. The experiment was terminated on day 56. (P, protein immunization; −ve, PBS formulated with adjuvant). **(B)** Quantification of serum-binding antibodies by ELISA over the course of the experiment. The antibody titers are presented for pooled sera at each time point. **(C)** Terminal neutralizing antibody titers against the matched virus from which the vaccines were derived. (ID_50_, half maximal inhibitory dilution). **(D)** Frequency of antigen-specific T cells recognizing SARS-CoV-2 peptide pools. (SFU, spot forming units).

The immunogenicity of the purified protein was tested in mice using a 3 μg inoculum, formulated in Alhydrogel^®^ (Figure 3A). S-binding antibodies were quantified with pooled sera to determine the endpoint titers after each immunization despite the low volume available for each bleed. Immunized mice developed robust binding antibodies that were detectable after the first immunization (Figure 3B). Binding antibodies plateaued after the second immunization and no obvious increase was observed after the final immunization. Reassuringly, negligible signal was observed in the control group comprising mice that were immunized with PBS formulated in adjuvant.

The frequency of IFN-γ secreting cells was quantified at the terminal endpoint using 2 overlapping peptide pools which spanned the length of the spike protein. Immunized animals developed robust responses against both peptide pools with responses of 990(±443) SFU/10^6^ splenocytes and 763(±492) SFU/10^6^ splenocytes for peptide pool A and B, respectively. No signal was observed for the control group which was immunized with adjuvant formulated in PBS. All immunized mice also developed high titers of neutralizing antibodies against the autologous virus at the terminal bleed (Figure 3C and Table 1) with titers ranging from 4,094 to 29,387 (median 7133.2). In one animal, the neutralizing activity was so high that 100% neutralization of the pseudovirus was observed at the highest dilution, and therefore, an exact titer could not be calculated. In the absence of a standardized assay, it is difficult to make comparisons between different studies or to easily relate the immunogenicity of these vaccines to those in clinical development – however, the high antibody titers elicited indicate significant immunogenicity of our vaccine.

**Table 1:** Summary of ID50 titers in mice immunized with the plant-produced S∆TM (vaccine) or immunized with PBS formulated with Alhydrogel^®^ adjuvant (−ve).

	Vaccine	−ve
Mouse #1	1368.7	0
Mouse #2	29386.8	4.9
Mouse #3	7133.2	0
Mouse #4	4094.9	0
Mouse #5	Out of range	0

## Discussion

The development of a plant-based production system that enables high yields of well-folded and appropriately glycosylated viral glycoproteins has the potential to result in a paradigm shift in vaccine production. However, differences in the plant cellular machinery compared to mammalian or other eukaryotic cell-based systems could undermine the efficient maturation of viral glycoproteins. This could result in low yields (Margolin et al., 2018), inefficient processing (Margolin et al., 2020c) and even aberrant glycosylation in the case of certain heavily glycosylated viral glycoproteins (Margolin et al., 2021). Engineering the secretory pathway provides a modular approach to address these limitations with the potential to produce complex biopharmaceuticals in plants that could not otherwise be made in sufficient quantities or in the appropriate conformation (Margolin et al., 2020b).

We have systematically investigated limitations along the secretory pathway to develop a suite of expression approaches to support glycoprotein-based vaccine development in plants (Margolin et al., 2020c, 2021). Following the emergence of SARS-CoV-2, we applied these approaches to a soluble spike antigen which served as a suitable example of a glycoprotein from an emerging virus. Consistent with our previous work (Margolin et al., 2020c), we observed a significant increase in accumulation when the spike was co-expressed with human calreticulin. The antigen was not in fact detectable under the conditions tested without co-expression of the chaperone, further reinforcing the belief that the plant chaperones may not efficiently support the folding of certain complex glycoproteins (Margolin et al., 2018). Integrating the co-expression of CRT with human furin supported the efficient cleavage of the protein *in planta* where the host machinery was unable to support this process. This highlights the malleable nature of the plant secretory pathway, which can be manipulated to improve the production of complex glycoproteins by the co-expression of multiple accessory proteins where necessary (Margolin et al., 2020b).

The co-expression of broad-spectrum protease inhibitors failed to appreciably improve the accumulation of the SARS-CoV-2 S∆TM antigen in this study. This is surprising given the impact this has had for several model proteins in previous work (Grosse-Holz et al., 2018). Based on this work, it is not possible to attribute any obvious effect to the endogenous plant protease repertoire, although we acknowledge that this has been well documented for recombinant antibodies and therefore remains an important outstanding question (Jutras et al., 2020; Puchol Tarazona et al., 2021). In this study, we were specifically interested in determining if the protease inhibitors tested had an additive effect to increase accumulation of the glycoprotein when co-expressed with calreticulin and therefore, the experimental design did not account for the potential impact these constructs may have during an extended purification. Further work would be necessary to address this question but if this was to prove true it would potentially enable cost saving by circumventing the need for supplementation of the extraction buffer with commercial protease inhibitors.

The recombinant protein was successfully purified but the yields were low, and gel filtration indicated that the antigen was highly heterogenous. Furthermore, protein aggregates predominated and there was no convincing evidence that well-folded trimeric protein was formed. Similar observations have recently been described for other plant-produced viral glycoproteins, where it was concluded that aberrant glycosylation may have compromised protein folding or resulted in unwanted aggregation (Margolin et al., 2021). Disappointingly, the yield of the recombinant antigen was too low to enable determination of the site-specific glycosylation and at present this remains an outstanding question. The low expression yields and heterogenous product observed may partly reflect the need for structure-based redesign of the antigen as coronavirus spikes typically express poorly – even in mammalian cells – without further stabilization of the prefusion trimer (Pallesen et al., 2017). The introduction of stabilizing proline mutations, in particular, has shown promise in improving coronavirus spike expression yields (Pallesen et al., 2017; Hsieh et al., 2020; Wrapp et al., 2020) and the latest designs have been reported to improve accumulation by more than an order of magnitude (Hsieh et al., 2020). Future work is planned to incorporate structure-based stabilization of the antigen with the plant expression platform to improve production yields as this is an important determinant of the suitability of the system to respond to pandemic outbreaks. This should also enable the determination of the site-specific glycan composition of the protein to investigate the impact of the host glycosylation machinery on the antigen. Glycosylation plays a central role in protein folding and aberrant glycosylation of plant-produced viral glycoproteins has been associated with unwanted aggregation (Margolin et al., 2021). The plant glycosylation machinery may also impart undesired plant-specific modifications to recombinant viral glycoproteins (Margolin et al., 2020b, 2021). Recent work by Shin and colleagues has further highlighted the importance of glycosylation in molecular farming where they showed that proper glycosylation is critical for production of a well-folded SARS-CoV-2 receptor binding domain (Shin et al., 2021). Low yields of viral glycoproteins remain a challenge for molecular farming (Margolin et al., 2018) and it is presently unclear if yields as high as cell culture systems can be achieved given reports of 10.5 mg/l of stabilized SARS-CoV-2 spike trimers (Hsieh et al., 2020).

Nonetheless, despite suboptimal production yields, sufficient antigen was recovered to conduct an immunogenicity study in BALB/c mice. The purified protein was highly immunogenic after low dose immunization, leading to the development of robust antibody and cellular responses. All immunized mice developed high titers of neutralizing antibodies against the sequence-matched pseudovirus and this is especially encouraging given the low vaccine dose used for immunizations. While the observed responses are highly encouraging, these are markers of vaccine immunogenicity rather than effectiveness and therefore, future work will investigate whether the plant-produced spike can protect against viral challenge in hamsters.

## Conclusion

In conclusion, this work comprises an important proof-of-principle for engineering the secretory pathway to produce vaccines against emerging viruses – and in particular for SARS-CoV-2. Future studies will need to integrate these approaches with structure-based vaccine design to ensure improved yields and the structure of the recombinant glycoprotein (Pallesen et al., 2017). Further work is also required to understand how the plant glycosylation machinery affects viral glycoprotein production in plants, particularly in the light of recent reports of aberrant glycosylation of heavily glycosylated viral targets (Margolin et al., 2021). Nonetheless, this study is highly encouraging and will inform further refinements to the production platform that is under development.

## Data Availability Statement

The datasets presented in this study can be found in online repositories. The names of the repository/repositories and accession number(s) can be found at GenBank, MN908947.3.

## Ethics Statement

The animal study was reviewed and approved by the University of Cape Town Animal Ethics Committee.

## Author Contributions

EM conceptualized the study with input from A-LW, RC, and ER. Protein production in plants was conducted by EM and MV, with supervision by AM. Immunogenicity assays were conducted by EM, WM, and GS. EM drafted the manuscript. All authors contributed to data analysis and reviewed the final manuscript before submission. Funding for the project was obtained by EM and A-LW.

## Funding

This research was funded in part, by the Wellcome Trust [203135/Z/16/Z]. For the purpose of open access, the author has applied a CC BY public copyright licence to any Author Accepted Manuscript version arising from this submission. Further support was provided by the South African Research Chairs Initiative of the Department of Science and Technology and the National Research Foundation (grant number: 64815). GS was supported by EDCTP2 programme (Training and Mobility Action TMA2018SF-2446).

## Conflict of Interest

EM, RC, AM, A-LW, and ER have filed a series of patent applications encompassing approaches to produce viral glycoproteins in plants, including for SARS-CoV-2: US 2019/0337994 A1, WO 2018 220,595 A1, PA174002_PCT, and PA2106659.4. AM and ER are shareholders in Cape Bio Pharms (SA) Pty. Ltd., a molecular farming company in Cape Town.

The remaining authors declare that the research was conducted in the absence of any commercial or financial relationships that could be construed as a potential conflict of interest.

## Publisher’s Note

All claims expressed in this article are solely those of the authors and do not necessarily represent those of their affiliated organizations, or those of the publisher, the editors and the reviewers. Any product that may be evaluated in this article, or claim that may be made by its manufacturer, is not guaranteed or endorsed by the publisher.
